# The advantage of intergenic regions as genomic features for machine-learning-based host attribution of *Salmonella* Typhimurium from the USA

**DOI:** 10.1099/mgen.0.001116

**Published:** 2023-10-16

**Authors:** Antonia Chalka, Tim J. Dallman, Prerna Vohra, Mark P. Stevens, David L. Gally

**Affiliations:** ^1^​ The Roslin Institute and R(D)SVS, University of Edinburgh, Edinburgh, UK; ^2^​ Institute for Risk Assessment Sciences (IRAS), University of Utrecht, Heidelberglaan, Utrecht, Netherlands

**Keywords:** host prediction, intergenic regions, machine learning, phylogeny, random forest, *Salmonella* Typhimurium

## Abstract

*
Salmonella enterica* is a taxonomically diverse pathogen with over 2600 serovars associated with a wide variety of animal hosts including humans, other mammals, birds and reptiles. Some serovars are host-specific or host-restricted and cause disease in distinct host species, while others, such as serovar *S*. Typhimurium (STm), are generalists and have the potential to colonize a wide variety of species. However, even within generalist serovars such as STm it is becoming clear that pathovariants exist that differ in tropism and virulence. Identifying the genetic factors underlying host specificity is complex, but the availability of thousands of genome sequences and advances in machine learning have made it possible to build specific host prediction models to aid outbreak control and predict the human pathogenic potential of isolates from animals and other reservoirs. We have advanced this area by building host-association prediction models trained on a wide range of genomic features and compared them with predictions based on nearest-neighbour phylogeny. SNPs, protein variants (PVs), antimicrobial resistance (AMR) profiles and intergenic regions (IGRs) were extracted from 3883 high-quality STm assemblies collected from humans, swine, bovine and poultry in the USA, and used to construct Random Forest (RF) machine learning models. An additional 244 recent STm assemblies from farm animals were used as a test set for further validation. The models based on PVs and IGRs had the best performance in terms of predicting the host of origin of isolates and outperformed nearest-neighbour phylogenetic host prediction as well as models based on SNPs or AMR data. However, the models did not yield reliable predictions when tested with isolates that were phylogenetically distinct from the training set. The IGR and PV models were often able to differentiate human isolates in clusters where the majority of isolates were from a single animal source. Notably, IGRs were the feature with the best performance across multiple models which may be due to IGRs acting as both a representation of their flanking genes, equivalent to PVs, while also capturing genomic regulatory variation, such as altered promoter regions. The IGR and PV models predict that ~45 % of the human infections with STm in the USA originate from bovine, ~40 % from poultry and ~14.5 % from swine, although sequences of isolates from other sources were not used for training. In summary, the research demonstrates a significant gain in accuracy for models with IGRs and PVs as features compared to SNP-based and core genome phylogeny predictions when applied within the existing population structure. This article contains data hosted by Microreact.

## Data Summary

A pipeline for broader adoption of the model building and testing is provided: https://github.com/Antonia-Chalka/stm_ml_pipeline. The authors confirm all supporting data, code and protocols have been provided within the article or through supplementary data files, as well as the github repository mentioned in the paper (https://github.com/Antonia-Chalka/stm_ml_pipeline). The training data can be specifically found at https://github.com/Antonia-Chalka/stm_ml_pipeline/blob/main/test_data/papertrainingdata.zip. All sequences used in this research were already publicly available in Enterobase or at NCBI. The full project numbers for every strain sequence analysed have been provided in Tables S1, S2 and S13, available in the online version of this article.

We have also uploaded the phylogenetic tree and related metadata on microreact (https://microreact.org/project/ftNtqnskJT4Jq332Z9G2AL-the-advantage-of-intergenic-regions-as-genomic-features-for-machine-learning-based-host-attribution-of-salmonella-typhimurium-from-the-usa).

**Impact Statement**

Machine learning models have been built that predict the farmed animal host of *Salmonella* Typhimurium strains based on their genome sequence. The present work includes a comprehensive comparison of different features extracted from isolate genome sequences and shows that models based on the regions between genes, intergenic regions, are the most accurate for host attribution. The work is important for public health to enable source attribution of *Salmonella* strains responsible for outbreaks. This is a particular challenge when pathogens have reservoirs in multiple hosts and can therefore be present in many types of food. The research is based on nearly 5000 genome sequences with development of a reproducible pipeline for generating the models along with their testing. The most accurate models predict that ~45 % of the human *S*. Typhimurium infections in the USA originate from cattle, ~40 % come from poultry and ~12.5 % from pigs. Our best-performing models clearly identify alternative host sources in the middle of the majority of host clusters and so represent an advance on use of the core structure alone. However, performance of the models was unreliable for isolates that fall outside of the phylogeny the models were trained within.

## Introduction

*Salmonella enterica* subspecies *enterica* is one of the leading bacterial causes of diarrhoeal and invasive disease worldwide [1, 2]. The World Health Organisation estimated that in 2010 non-typhoidal *S. enterica* serovars caused 78 million human illnesses resulting in the loss of over 59 000 lives and 4 million disability-adjusted life years worldwide. Most human infections are foodborne and farmed animals are key reservoirs of *S. enterica *.

There are over 2600 antigenically distinct serovars of *S. enterica *, which differ in host-specificity, tissue tropism and virulence [3]. Generalist serovars are also prominent in outbreaks. In England and Wales, Public Health England reported 8630 laboratory-confirmed cases in 2016, of which 2356 were due to Enteritidis serovars and 1700 were due to Typhimurium [4]. Similarly in Scotland, Health Protection Scotland identified 756 cases of human non-typhoidal *Salmonella* in 2019, with 58 % of cases caused by Enteritidis or Typhimurium serovars [5]. *Salmonella* Typhimurium (STm) is one of the most common *Salmonella* serovars responsible for foodborne infections in the EU [6], UK [4, 5] and USA [7] and this partly reflects its capacity to colonize a wide range of animal hosts, such that zoonotic transmission occurs.

Though a significant aspect of combating *S. enterica* outbreaks is determining the reservoir of origin, much of the genetic basis of host-specificity and zoonotic risk remains ill-defined. Resolving serovar host-specificity is further complicated by the existence of subgroups, or pathovariants, within serovars that differ in tropism, as shown by the existence of host-adapted variants causing invasive non-typhoidal salmonellosis [8]. Subgroups within the STm serovar also differ in host tropism [9], such as the human-adapted sequence type (ST) 313 strains associated with invasive typhoid-like disease in sub-Saharan Africa [10, 11] and definitive phage types (DTs) 40, 56 and 160 in wild passerines [12].

When food-based outbreaks of human salmonellosis occur, rapid attribution of the likely animal source of the infection may help inform the common food vehicle transmitting the pathogen, allowing interventions to prevent further cases. Considering that serovars with a broad host range cause almost half of *Salmonella* infections in humans in the UK and other countries, improved methods for source attribution during outbreak investigations are needed. This, in turn, requires a better understanding of the genetic basis of host-specificity in *S. enterica *, including identification of features associated with zoonotic risk or elevated virulence and to understand how gain or loss of features may affect these. Screening of random STm mutant libraries across chickens, pigs and cattle has revealed that while a core set of genes is important for intestinal colonization of all three farmed animal species, smaller gene sets play a role in persistence and virulence [13], although how these relate to colonization and disease severity in humans is largely unknown.

Machine learning (ML) models are able to detect patterns in complex data, and have increasingly been used on ‘omics’ datasets to investigate complex genomics questions [14]. In relation to *Salmonella* host-specificity, ML models have been applied to predict functional variations in protein-coding genes associated with invasive disease [15], as well as for source prediction across *Salmonella* serovars Dublin, Typhi and Typhimurium [16–18]. Lupolova *et al.* [16] built Support Vector Machine classifiers based on protein variants (PVs) to predict the source (human vs bovine vs swine vs avian) of STm sequences using gene presence and absence data with marked differences across hosts. Their phylogenetic clustering based on accessory genome content indicated the presence of specific subclusters in their dataset that were host-restricted, particularly a large avian cluster. The resulting models led to the conclusion that there was a marked host restriction in the STm dataset analysed, particularly in the human-derived isolates, with only specific subsets of isolates being capable of infecting different hosts. In a broader approach, Zhang *et al.* [18] analysed sequences from a wider diversity of STm isolates from human, bovine, poultry, wild birds and swine. They built their source-prediction Random Forest models from sequences of isolates from farmed animals, using insertions or deletions, SNPs and accessory genes as predictive features. Their resulting model had good accuracy and precision for animal attribution although attribution for human isolates was difficult with the majority (68.1 %) not attributed as zoonotic in origin which they consider is due to needing more data of STm isolates from other sources including the environment [18]. Their model did correctly identify the source of 7/8 zoonotic outbreaks [18]. Difficulty in ascribing ‘original’ sources for human-derived STm isolates in the work of Zhang *et al.* [18] relative to that of Lupolova *et al.* [16] may be a result of how the models were built (including vs excluding humans as a predicted host), and the diversity of the datasets as defined in the former [18], illustrating the importance and care required in building a high-quality dataset that will result in models that yield reliable predictions. Additionally, a recent study by Munck *et al.* [17] applied a Danish STm dataset for source prediction based on core genome multilocus sequence type (cgMLST) features, taking extra care to account for the difference in distribution of sequences across their host when fine-tuning their model.

Enabled by advances in computational power, proliferation of ML frameworks and availability of increasingly large datasets of bacterial whole genome sequences, we aimed to dissect and predict host association in STm. Specifically, we demonstrate advancement in this field by: (1) building STm host-predictive models by using larger training and testing datasets and applying an expanded predictor feature set – PVs, intergenic regions (IGRs), antimicrobial resistance (AMR) profiles and SNPs; (2) creating a pipeline for reproducible generation and testing of models; (3) comparing the performance of ML host predictions against phylogeny-based host predictions; (4) examining the features implicated in STm host association by ML models; and (5) validating the best models with an ‘unseen’ USA STm strain set. We intend to work with public health agencies in the USA and UK to integrate this methodology into outbreak investigations for STm.

## Methods

**Fig. 1.**
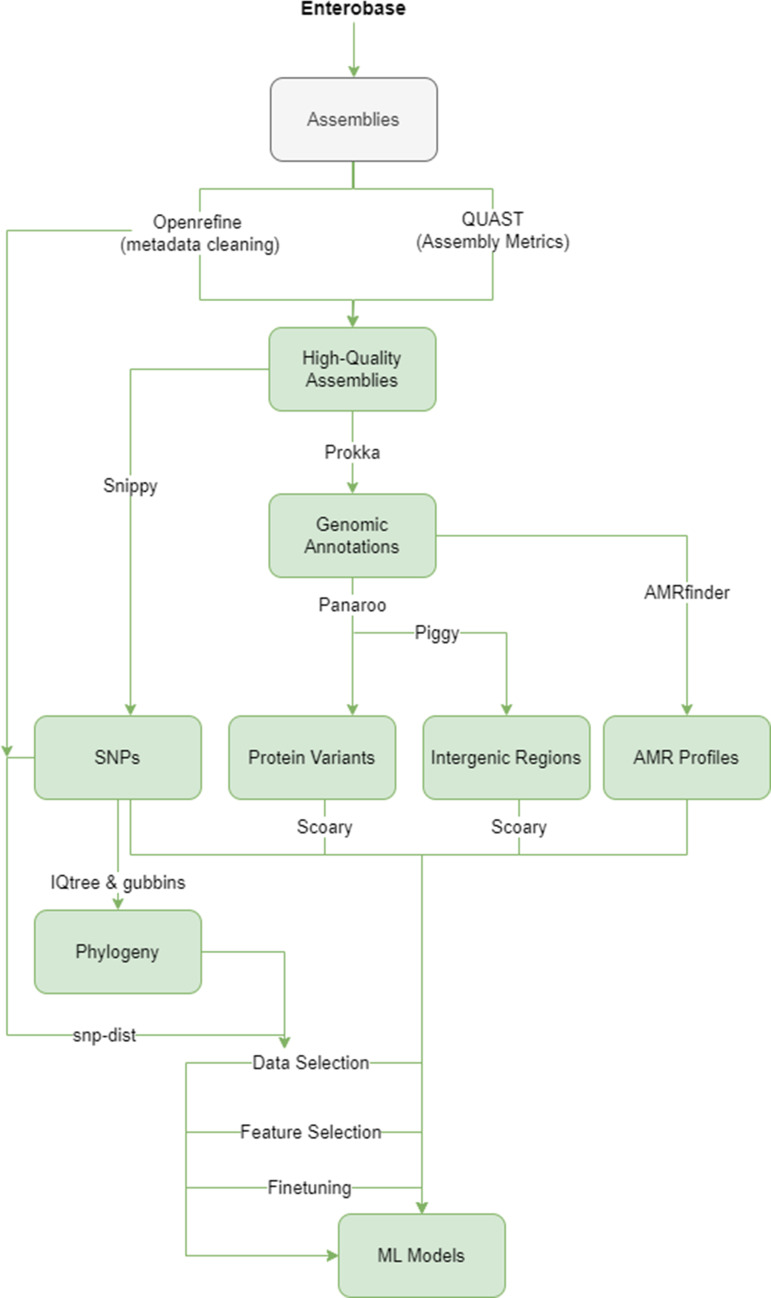
Simplified flowchart of the process used for model creation, which has been adapted as a Nextflow pipeline. Input assemblies with associated metadata were filtered, annotated and their genomic features extracted to build several machine learning models.

**Fig. 2.**
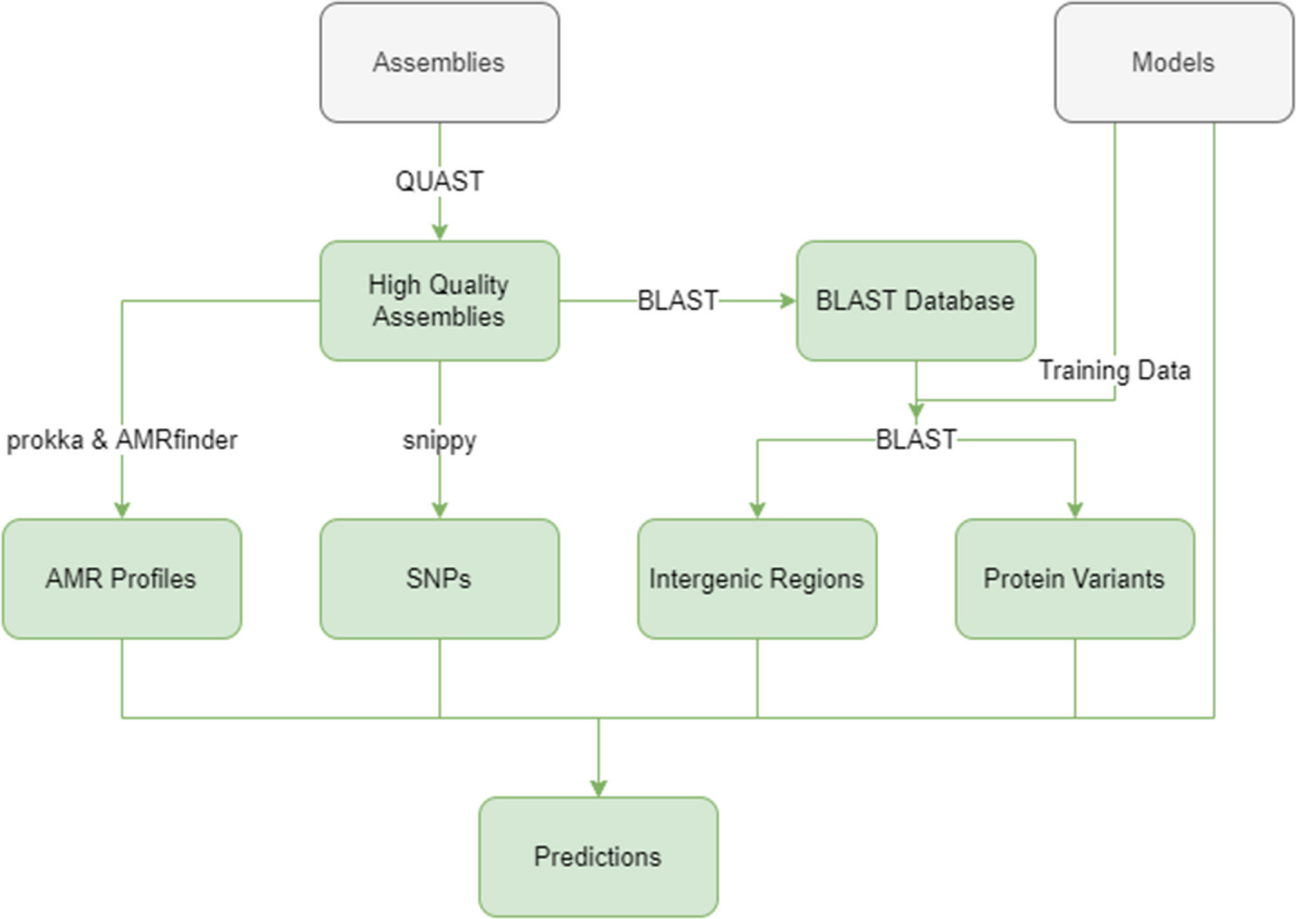
Simplified flowchart of inputting new assemblies for host-prediction based on pre-existing models.

The workflows for gathering high-quality assemblies, metadata, genome annotation and extraction of features to build and implement ML models are summarized in Figs 1 and 2. All information about the versions and parameters used for each tool and for hyperparameter testing is found in release 1.0 of our pipeline. The following software was used:

- quast:5.0.2
- prokka:1.14.5
- ncbi-amrfinderplus:3.10.5, run by inputting both protein and nucleotide files (-p, -g), and run with the options --plus and -O Salmonella
- piggy:1.5, run with options: -l 90 n 90
- panaroo:1.2.9, run with options: --remove-invalid-genes --merge_paralogs --clean-mode moderate
- snippy:4.6.0
- mlst:2.23.0
- tidyverse:4.0.5 (R package)
- scoary:1.6.16
- snp-dists:0.8.2
- blast:2.12.0
- seqtk:1.3
- C50 : 0.1.8 (R Package)
- caret:6.0–94 (R Package)

### Data collection

Enterobase [9, 19] was queried in October 2019 for STm sequences with associated host data that fulfilled the following criteria:

- Known host (either human, bovine, poultry or swine)
- Collected from 1998 onwards
- Isolated in the USA

The above criteria were implemented to reduce geographical bias and reflect recent STm genomes present in the population. In total, 5200 STm assemblies (Tables S1 and S2) were initially collected. Openrefine [20] was used to standardize metadata.

**Table 1.** Number of STm assemblies used in model creation, divided by host and sequence type Three datasets were formed to create the following types of models: ‘All’ – models created from a non-clonal dataset of all hosts (human, bovine, swine, poultry) to identify the host of an unknown isolate; ‘BPS’ (bovine, poultry, swine) – models created from a non-clonal dataset that excluded human-derived assemblies primarily in order to attribute an animal source to human-derived isolates; and ‘Human’ – models created from a non-clonal dataset where hosts were defined as either human or livestock, with the aim of assigning a ‘human’ host score for any animal isolate. Ideally, such scoring could be used to assess the zoonotic risk of a livestock isolate.

Sequence type	Host	Count
128	Human	1
128	Poultry	1
128	Swine	1
19	Bovine	281
19	Human	1749
19	Poultry	1268
19	Swine	358
2066	Poultry	1
213	Human	27
213	Swine	8
2379	Bovine	1
2379	Human	6
2379	Poultry	8
2379	Swine	2
34	Bovine	10
34	Human	105
34	Poultry	8
34	Swine	45
99	Human	3

**Table 2.** Summary of model performance The dataset and feature-set used for each model are listed alongside their counts. For accuracy, the Kappa statistic calculated using the testing set is used.

Dataset	Assembly count	Features used	Feature counts	Accuracy (Kappa)
All	3383	PV	854	0.93
All	3383	IGR	999	0.98
All	3383	SNP	1507	0.67
All	3383	AMR Gene	115	0.74
All	3383	AMR Class	17	0.71
BPS	1992	PV	854	0.93
BPS	1992	IGR	999	0.99
BPS	1992	SNP	1507	0.78
BPS	1992	AMR Gene	115	0.63
BPS	1992	AMR Class	17	0.60
Human	3383	PV	854	0.94
Human	3383	IGR	999	0.98
Human	3383	SNP	1507	0.66
Human	3383	AMR Gene	115	0.75
Human	3383	AMR Class	17	0.74
All	3383	Phylogeny	na	0.75

#### Assembly quality filtering

To ensure only high-quality assemblies were used to create the models, assembly metrics were calculated with QUAST [21]. Assemblies were filtered using the following thresholds, which were based on quality assessment of whole-genome *Salmonella* sequences [22]:

- Total assembly length between 4 and 6 Mb
- <500 contigs
- Largest contig >100 kb
- N50 >50 kb
- GC content between 50 and 54 %

#### Clonal detection

To assess the presence of closely related assemblies in the dataset that were epidemiologically linked, SNP distances were calculated using snp-dists [23]. Two or more assemblies were clustered and classified as a clonal group if they fulfilled all of the following conditions:

- <10 SNP difference
- Same host
- Same state
- Same year

With the above information, a non-clonal filter was implemented. From each resulting clonal cluster, a representative was randomly chosen, and the rest were discarded to create a non-clonal STm dataset from which training sets were extracted to build the models.

#### Dataset creation

MLST [24, 25] was used to assign sequence types. From the non-clonal STm dataset, 3883 assemblies were used as training and testing data (Table 1). Ten additional ST313 assemblies were used for further testing.

### Genomic feature extraction

High-quality assemblies were annotated with Prokka [26]. A nucleotide FASTA file of reviewed STm proteins from UniProt [27] was inputted into Prokka using the ‘trusted protein file’ option to ensure consistency between gene names across annotated assemblies.

AMRfinder [28] was used to extract AMR-specific genes. Snippy [29] was used to extract core SNPs with STm strain SL1344 as the reference genome [30]. Panaroo [31] was used for pan-genome extraction, alongside Piggy [32] for clustering intergenic regions in a pan-genome-like fashion.

As a large number of predictor features across the IGR, PV and SNP datasets would increase the chances of overfitting, a correlation-based filtering step was implemented. Scoary [33] was used to calculate association scores for IGRs and PVs across all hosts, and was run with the ‘--collapse’ parameter, which grouped features that were identically distributed across samples. Features that had a statistically significant association (Bonferroni-corrected *P*<0.05) with at least one host were kept in the dataset, whereas features with no statistically significant association with any of the hosts were discarded. SNPs were filtered for >0.1 % and <99.9 % abundance from the dataset.

The following feature datasets were created:

- AMR Class: presence/absence matrix of the class of antibiotic resistance genes present in an assembly
- AMR Gene: presence/absence matrix of AMR genes identified by AMRfinder within an assembly
- IGR: presence/absence matrix of IGRs that were found to be statistically associated with at least one host (*P*<0.05, Bonferroni correction)
- PV: presence/absence matrix of predicted PVs that were statistically associated with at least one host (*P*<0.05, Bonferroni correction)
- Core SNPs: core SNPs, filtered for >0.1 % and <99.9 % abundance from the dataset.
- An additional set of SNPs were filtered using scoary, similar to PVs and IGRs, and used to generate an additional set of SNP models, and their performance and metrics are given in Tables S10 and S11.

### Phylogenetically based prediction

Core SNPs identified with Snippy were used to build a phylogenetic tree via Gubbins [34] and IQ-Tree [35] of all the STm assemblies that passed the quality filter. Hierbaps was used to assign the assemblies into Baps clusters [36, 37]. For the phylogeny-based host assignment, models based on 1–5 closest neighbours were created (*k*=1–5). For the *k*=1 phylogenetic models, the host of the closest neighbour based on branch length was used as a predictor (Table S6). For *k*=2+ models, the most commonly occurring host was selected as the prediction. In case of a tie, the host with the shortest average branch length was selected.

### Model generation

Random Forest was the chosen ML method due to its performance being comparable to other algorithms in similar datasets [38], its ability to investigate non-linear interactions between features, its resistance to overfitting and the ease of extracting important features for future investigations. PVs, IGRs and both AMR datasets were inputted as a presence–absence matrix (0/1), whereas SNPs were inputted with their corresponding base (A/T/G/C) per position. Models using the ‘All’ and ‘BPS’ datasets were multiclass classifiers, which would predict either human/bovine/poultry/swine (‘All’ dataset-based models) or bovine/swine/poultry (‘BPS’ dataset-based models). Models built using the ‘human’ dataset were binomial classifiers (human/livestock). An additional set of models were trained using Leave Group Out Cross Validation (LGOCV) based on the BAPS clusters to test the effects of missing phylogeny.

Models were built in R using the Caret [39] and C50 [40] packages, with 10-fold cross-validation, repeated three times and with a 75/25 split of training/testing data. Fine-tuning was automatically handled by Caret, with ‘trials’ and ‘winnow’ tuned separately for each model. The overall performance of the models was judged using the Kappa statistic (also referred to as Cohen’s Kappa) which measures how closely the host’s assignment by the ML classifier matched the actual observed hosts, controlling for an ‘accuracy’ derived from random guessing (i.e. expected accuracy). Kappa = (observed accuracy − expected accuracy)/(1 − expected accuracy).

Typically, when outputting a prediction for a single sample for a classification Random Forest model, each decision tree in the model will output a ‘vote’ for a single class. To make a final prediction, the model outputs the class with the majority vote as the prediction, as well as the proportion of that vote as a confidence score. As the dataset was imbalanced, receiver operating characteristic (ROC) curves were used to fine-tune the thresholds for the confidence score used for predicting the host (threshold moving) [41]. The resulting thresholds were used for host-calling in lieu of the default method of using the host with the highest score. For each model, an ROC curve was calculated, which plotted the true positive rate vs the false positive rate. The area under the curve (AUC) of the ROC curve was calculated to provide an additional aggregate measure of model performance that allowed for fine-tuning of the thresholds used for the final prediction. Compared to the default thresholds (~0.25 for models built using the ‘All’ dataset, ~0.33 for models built using the ‘BPS’ dataset, ~0.5 for models built using the ‘human’ dataset), the optimal thresholds differed in the ‘All’ models (higher thresholds for human predictions, lower thresholds for bovine/swine) and the BPS models (higher thresholds for poultry, lower for bovine), but were similar in the ‘human scoring’ models (Table S12). The full list of predictions for each sequence of each model can be found in Table S7.

Feature importance was judged using the ‘overall’ metric, which is determined by the percentage of training set samples that pass through a feature’s node in the tree [40], with the predictor at the root/first split of the tree having an importance score of 100 %.

Enterobase was queried and an additional 244 recent (2019–2022) USA STm sequences were collected (Table S13) as a test set within the USA STm population structure. Assemblies were inputted into the testing pipeline, in which PV/IGR presence/absence was determined by comparing the assemblies against a multifasta file containing every unique allele in an IGR/PV cluster via blast (Fig. 2).

Further analysis of the data and generation of figures was performed in R. The full list of scripts and packages applied can be found in the github repository.

### Pipeline implementation

#### Model building

The steps for building host-prediction models were implemented in a DSL2 Nextflow [42] pipeline, with the option of using Docker [43]/Singularity [44] containers, and hosted in a Github repository (https://github.com/Antonia-Chalka/stm_ml_pipeline). The pipeline requires a set of assemblies as well as a metadata file containing at least the filename and host, with optional fields being region and year, which would enable non-clonal filtering. Currently, the pipeline implements all of the previously described steps for model creation, with two exceptions: the generation of a phylogenetic tree due to its computationally expensive nature, and threshold moving for testing new data (Fig. 1). Additional data (R scripts, reference genomes, trusted protein file) are provided but not hard-coded, allowing the user to adjust them for their use-case.

#### Model testing

In addition to model creation, a ‘model testing’ pipeline was created to test existing models on a new dataset of assemblies, and is included in the Github repository. The process of creating the model testing pipeline was similar to that used for model creation, with one major point of divergence: the PVs and IGRs used to train the model were aligned to the new assemblies by blast, ensuring consistency of genetic features (Fig. 2). The resulting features were then run through their respective model, and the host predictions were outputted as a .csv file for further use.

## Results

### Comparison of the accuracy and precision/recall of the different Random Forest models

Of the 5200 genome assemblies for STm isolates from the USA obtained from Enterobase, 4809 passed the assembly quality thresholds, and 3883 passed the non-clonal filter as defined in the Methods. The final set of 3883 STm assemblies was used for model training and testing (bovine=292, human=1891, poultry=1286, swine=414). Based on the *S. enterica* MLST, seven STs were present in the dataset (Table 1) with most belonging to ST19 (*n*=3656), followed by ST34 (*n*=168). Table 2 provides the Kappa statistic for each of the different models built from the three main datasets (All, BPS and Human, as defined in the Methods) each with either PVs, IGRs, SNPs, AMR gene or AMR class as features. It was not anticipated that models built with AMR characteristics would perform as well as those based on more general information (PVs, IGRs, SNPs) but were included as they may identify AMR alleles/classes that were discriminatory between hosts.

Models based on PVs and IGRs performed best across all the different models (Table 2), reaching Kappa values of 94–99 %, followed by SNPs and AMR gene/class (Table S10). Though both IGRs and PVs had very high accuracy scores, IGRs consistently outperformed PVs. For example, in the main ‘All’ models that assign isolates to either swine, avian, cattle or human sources, the Kappa statistic was 0.983 for IGRs and 0.930 for PVs, which significantly outperformed SNPs (0.673).

The models were interrogated further for their combined precision and recall as indicated by the F1 score (Table S11). Bovine-derived assemblies were the most difficult to predict correctly (Table 3), with mis-attribution as human or swine. The effect was more prominent in the SNP-based models which often attributed the host of bovine-derived STm assemblies as human, or more rarely swine, resulting in the lower accuracies observed in Table 2. SNP models performed better when asked to disambiguate between assemblies of isolates from farmed animals in the ‘SNP, BPS’ model.

**Table 3.** F1 scores of the prediction of different hosts across models

Model (Feature, Dataset)	Bovine	Human	Poultry	Swine
AMR Class, All	0.22	0.84	0.87	0.61
AMR Gene, All	0.42	0.85	0.89	0.69
IGR, All	0.86	0.98	0.98	0.90
PV, All	0.78	0.95	0.97	0.88
SNP, All	0.06	0.78	0.90	0.70
AMR Class, BPS	0.23	na	0.90	0.68
AMR Gene, BPS	0.41	na	0.91	0.75
IGR, BPS	0.88	na	0.98	0.91
PV, BPS	0.91	na	0.99	0.94
SNP, BPS	0.78	na	0.95	0.87
AMR Class, Human	na	0.83	na	na
AMR Gene, Human	na	0.84	na	na
IGR, Human	na	0.95	na	na
PV, Human	na	0.98	na	na
SNP, Human	na	0.80	na	na

**Fig. 3.**
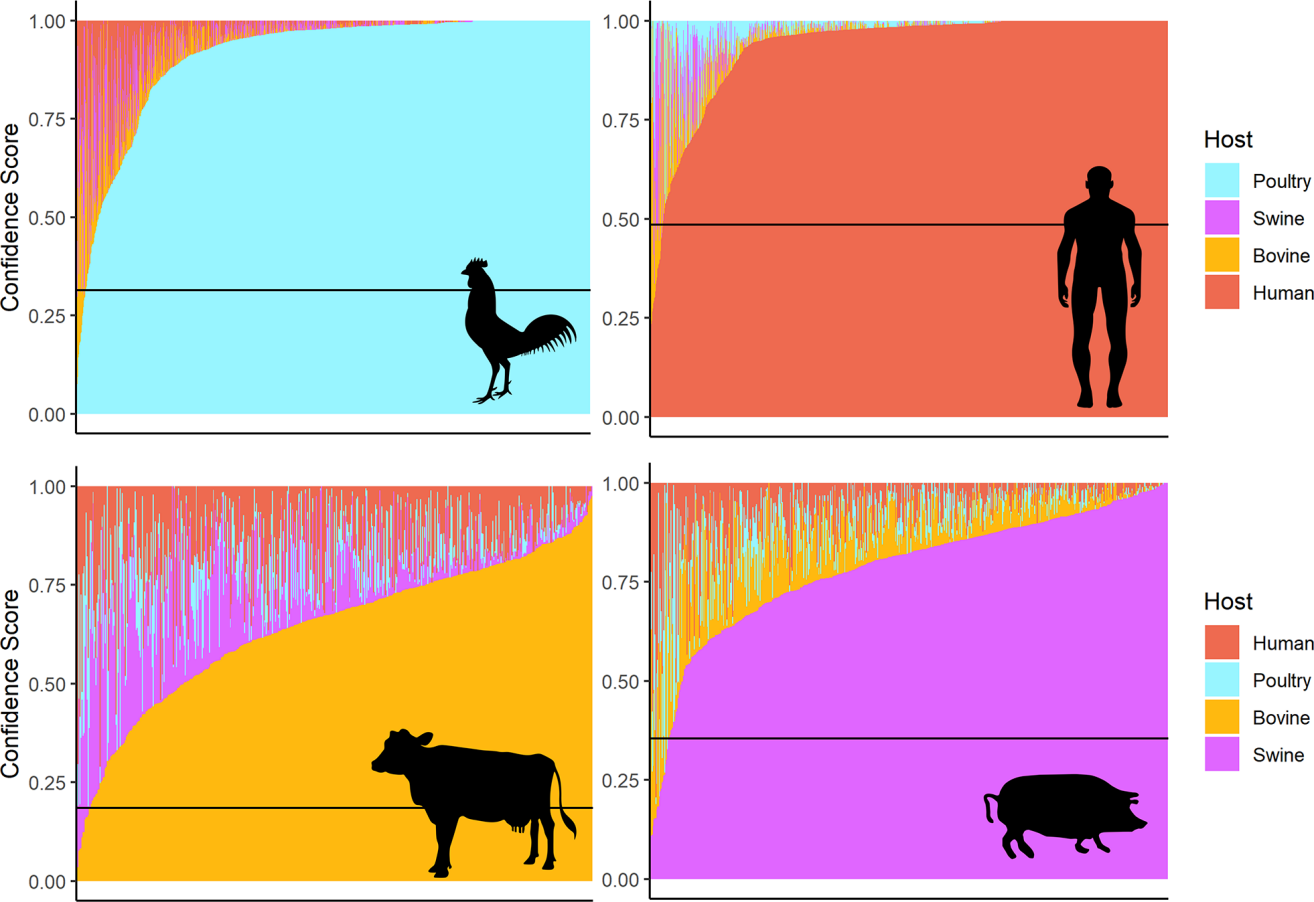
Chart of stacked confidence scores for each host in the 'IGR All' model, chosen as a representative. Each chart contains the STm isolates from the host pictured in its right-hand side. The stacked bars represent the confidence score for each of the four possible hosts (bovine, swine, poultry, human) for a single isolate (totals to one for each isolate). The horizontal line represents the thresholds set by the ROC curve.

The difficulty in predicting between bovine and swine as the host of origin of STm isolates was reflected in the overall confidence scores and prediction thresholds across the models (Fig. 3). While most human- and avian-derived isolates had high confidence scores, swine- and bovine-derived assemblies had more strains with reduced confidence.

### Analysis of the prediction models in the context of phylogeny

**Fig. 4.**
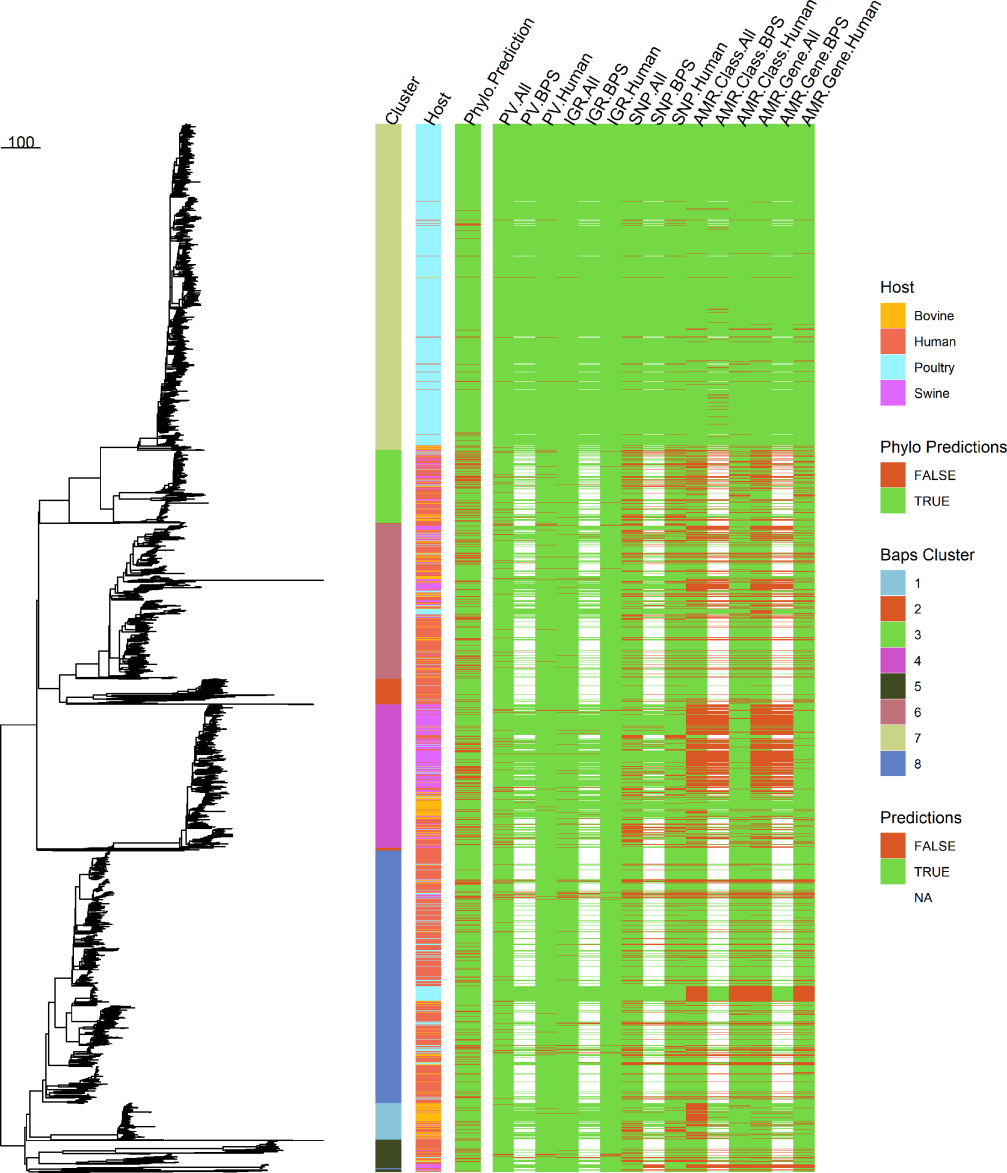
Core genome phylogenetic tree, BAPS cluster, source host and prediction accuracy of all 4809 (passed QC but not non-clonal filter) STm assemblies. Predictions were coloured green if correct, red if incorrect. An NA prediction only applies to BPS models which were trained to predict only farm animal hosts, and thus by their very nature could not ‘correctly’ predict human-derived STm assemblies. Scale bar represents substitutions per site. These data can be viewed at: https://microreact.org/project/ftNtqnskJT4Jq332Z9G2AL-the-advantage-of-intergenic-regions-as-genomic-features-for-machine-learning-based-host-attribution-of-salmonella-typhimurium-from-the-usa.

A maximum-likelihood phylogeny of the 4809 STm assemblies that passed the assembly quality thresholds was reconstructed using core SNPs (Fig. 4). Bayesian Analysis of Population Structure (BAPS) divided our dataset into eight major clusters. Most prominent is the large poultry cluster 7 which contained sporadic instances of human- and bovine-derived STm assemblies. Cluster 8 is primarily composed of human-derived assemblies, though with many interspersed assemblies from other hosts. Cluster 4 is made of up of mostly swine-derived assemblies though with a distinct bovine sub-cluster. Other clusters are more intermixed between the different hosts.

To determine the value of ML prediction over a phylogenetically based approach, a simple phylogenetically based prediction method was devised, using the closest neighbour of an assembly to assign a host. The phylogenetically based prediction had a Kappa statistic of 0.75, which is equivalent to the performance of the SNP- and AMR-based models but underperforms when compared to the PV or IGR models (Table 2). Additional nearest-neighbour phylogenetic models were generated (*k*=2–5), but their Kappa statistics were lower (0.69–0.63).

The prediction accuracy for all models, including the one based on phylogenetic analysis, was plotted against the phylogenetic tree for comparison, which included all STm assemblies that passed the quality filtering step (Fig. 4). Certain assemblies within the tree proved challenging for all models to predict. Many of these assemblies were ‘edge cases’ in clusters with one host vastly over-represented (e.g. human-derived assemblies in the poultry-abundant cluster 8). PV- and IGR-based models, besides generally performing better than prediction based on phylogenetic analysis, were able to correctly predict the host of many, though not all, such cases. For example, there were 63 non-poultry isolates spread across BAPS cluster 7. Despite being in the same clade with 1434 poultry-derived isolates, the ‘IGR, All’ and ‘PV, All’ models correctly assigned 55 and 51 of those isolates to their source host respectively, whereas the nearest-neighbour and the SNP-based models incorrectly called the majority (*n*=39) of these. In contrast, BAPS cluster 3 is a highly heterogeneous group and serves as a showcase for the IGR-based models outperforming the PV-based ones. The ‘IGR, All’ model called the majority of the isolates correctly, with 14 out of 334 isolates being predicted as the wrong host. In comparison, the ‘PV, All’ model called 33 isolates incorrectly. It is also notable that the added sensitivity conferred by IGRs over PVs is not necessarily consistent across all BAPS sub-clusters. In contrast to aforementioned cluster 7, the incorrect predictions made by the ‘IGR, All’ and ‘PV, All’ models in cluster 6 were for different sets of strains.

A concern when generating such host attribution models is the reliability of the models on new data and dependence on phylogeny. Therefore, in addition to validating models using new data, it is useful to assess how the models created using the approach and dataset employed by this paper perform when tested on data that fall outside of the training dataset phylogeny. For this, we employed an LGOCV method to build an additional set of models. In this process, the dataset is divided into a training subset, which includes all BAPS clusters except one, and a testing subset, which is based on the excluded cluster. This procedure was repeated to generate models of each feature and type combination, for each excluded cluster. By using the excluded clusters to test the model, one can simulate and evaluate the effect of ‘missing’ phylogeny on model performance.

**Fig. 5.**
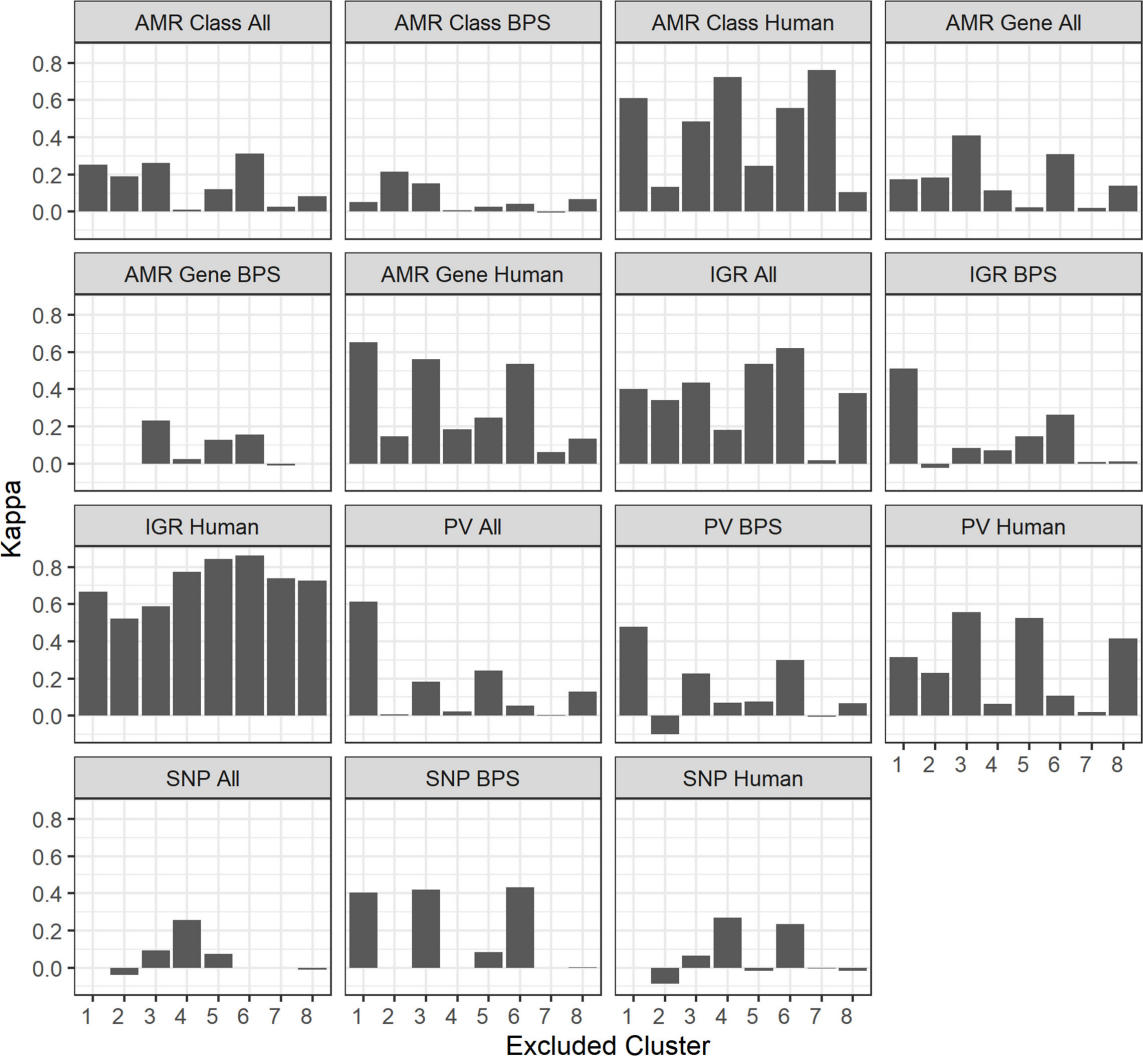
Performance of LGOCV models based on BAPS clustering. Results are split by model type. The excluded cluster indicates which cluster was removed from the training dataset and used for testing only. The Kappa statistic was applied as a measure of accuracy and is based on the model predictions with excluded cluster isolates only. A negative Kappa value indicates that the models’ attributions were worse than if predictions were random.

Fig. 5 illustrates that when these models were tested on BAPS clusters that were not part of their training data, the performance of the models varied greatly. While some models, such as ‘IGR Human’, had relatively high prediction accuracy no matter which cluster was excluded during the training process, and LGOCV models based on IGR and PV features generally exhibited higher Kappa values than those based on AMR or SNP values (except for the ‘AMR Class Human’ model), the effect was not consistent and model performance varied. There is also a general but not consistent trend that LGOCV models based on distinguishing between human and livestock sequences performed better than those trained to distinguish between livestock hosts. Cluster diversity may have some effect on model performance; for example, models created by excluding the large poultry cluster 7 were very poor at correctly assigning the excluded poultry isolates. However, much like feature or model type, that effect was not consistent.

### Prediction of the host of origin of human-derived STm isolates

**Fig. 6.**
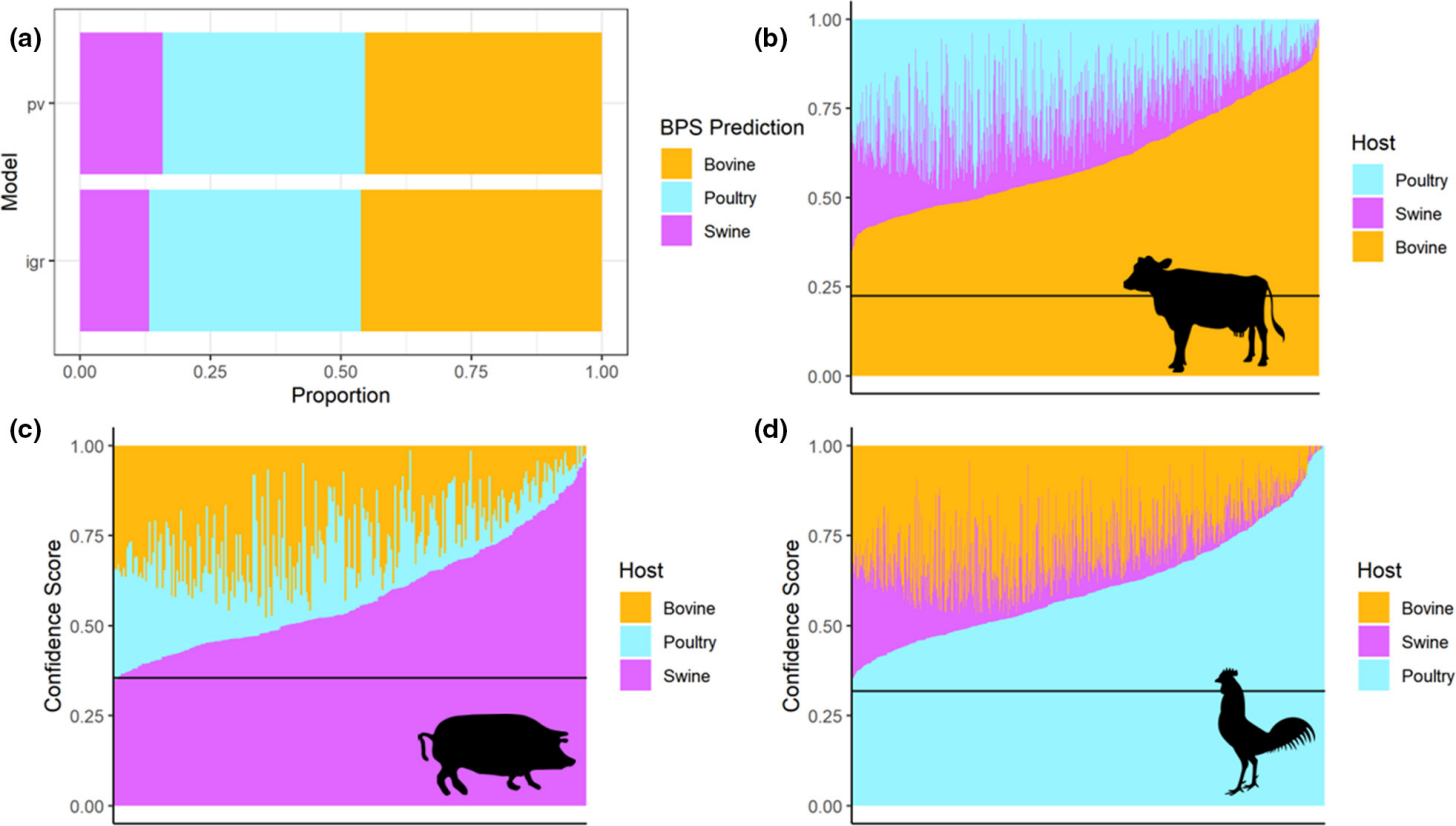
**(a**) Proportion of livestock host predictions for human STm assemblies in the IGR and PV BPS models. (b–d) Charts of stacked confidence scores of the human-derived STm assemblies passed through the 'IGR, BPS’ model. Each chart only contains the human-derived isolates that were predicted to originate from a specific livestock host, pictured at the right-hand side (b: bovine, c: swine, d: poultry). The stacked bars represent the confidence score for each of the three possible livestock hosts for a single isolate (totals to one for each isolate). The horizontal line represents the thresholds set by the ROC curve.

Human-derived STm assemblies were passed through the BPS models, which only assign isolates to livestock hosts (poultry, bovine and swine), to estimate the possible relative attribution of human infection from these established sources (Table S8). The results of the best-performing model (IGR) are shown in Fig. 6. Across the models based on the different features, most human-derived STm assemblies were predicted to originate from bovine, followed by poultry and swine.

Models with lower accuracies such as AMR- and SNP-based models (Table 2) predicted more assemblies originating from poultry. The best-performing model, ‘IGR, BPS’, predicted that 40 % of the human-derived assemblies originate from poultry, 45 % from bovine and 14.5 % from swine. Nearly all (99 %) our human STm isolates could be accurately attributed to a livestock source (highest confidence score exceeding the threshold set by the ROC curve), suggesting the BPS models were able to identify livestock-specific signals in the human-derived STm isolates.

### Prediction of the zoonotic risk of STm isolates found in livestock

As a form of validation, ten ST313 assemblies were inputted into the human vs livestock (BPS) models, of which 9/10 were predicted as originating from human across the different model types (Table S9). The PV-based models assigned the ST313 isolates as human with a confidence of 0.76–0.82, whereas the IGR-based model gave a more conservative 0.56–0.67 confidence score for human assignment, both well above the 0.5 threshold.

**Fig. 7.**
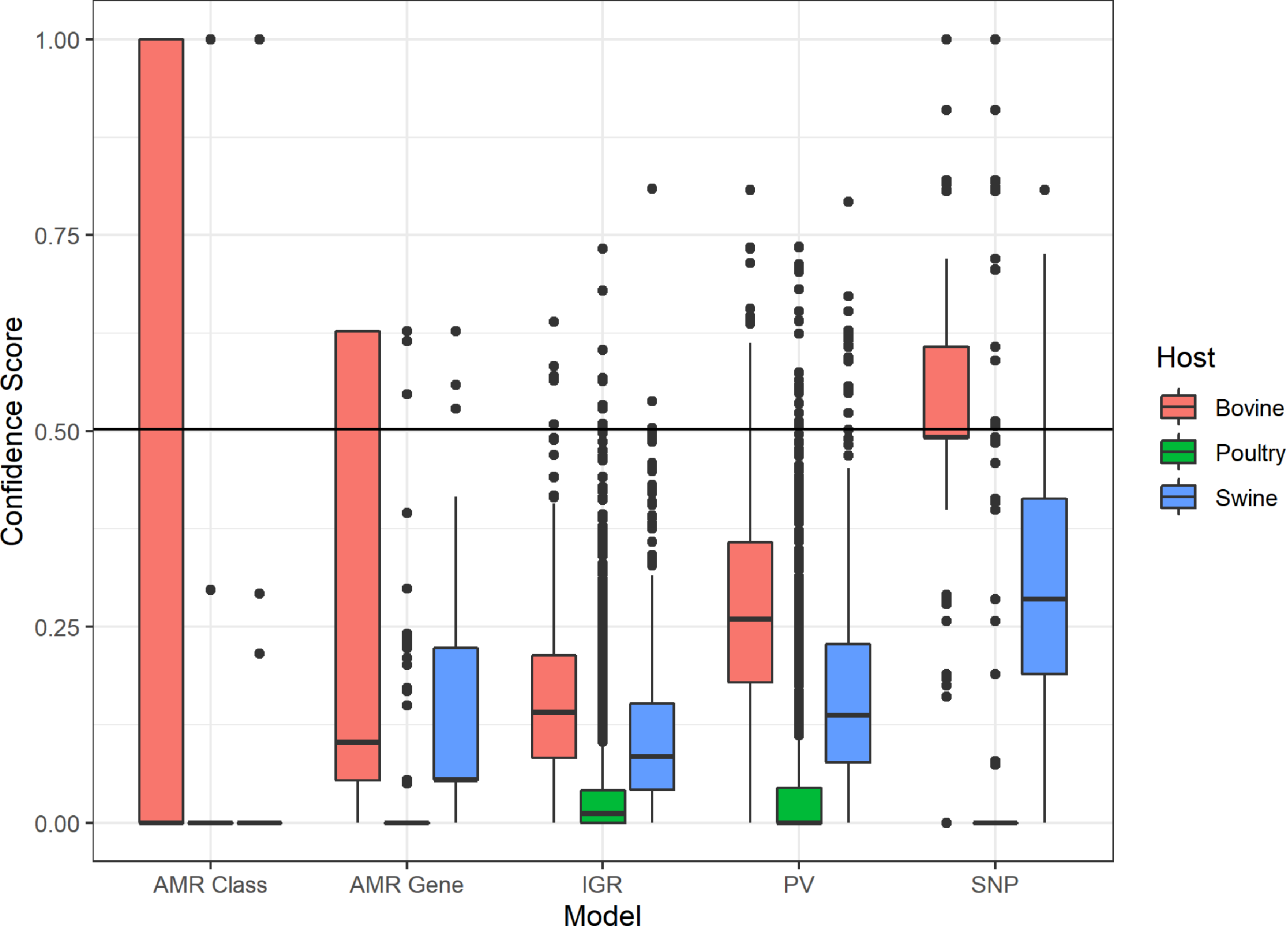
Boxplot of human threat prediction for farm animal-derived STm assemblies. The horizontal line represents the average threshold required for human host prediction (0.49–0.51) across all models.

Additionally, the farm animal-derived assemblies from our STm dataset were inputted into the human vs livestock models that predict a human host score and are potentially indicative of threat of human infection (Fig. 7). In the most reliable models (PVs and IGRs), the bovine isolates had a higher median score than the porcine or poultry isolates. It was notable that for all three livestock sources, there were a relatively low number of isolates (swine=20, poultry=28, bovine=38 for the ‘PV, Human’ model; swine=3, poultry=10, bovine=6 for the ‘IGR, Human’ model) that had a confidence score of >0.5, which is above the ROC-calculated threshold for a ‘human’ prediction.

### Validating model predictions on new isolate sequences from livestock

A set of 244 USA STm sequences (bovine=29, poultry=181, swine=34) collected between 2019 and 2022 were inputted into the PV and IGR models to simulate a real-world application of using the models to predict the host of new strains identified in the USA (Table S14). All models were able to predict the majority of the hosts correctly. BPS models performed better than the All models, and IGR models had generally higher accuracies than PV models (Table 4).

**Table 4.** Prediction accuracy of testing set of BPS assemblies across the best performing (PV and IGR) models

Model	Accuracy	Kappa
PV All	91	81
PV BPS	93	84
PV Human	99	na
IGR All	93	84
IGR BPS	95	87
IGR Human	100	100

### Identification of potentially significant features from high scoring features of the better performing models

Due to their good performance and ease of investigation, PVs with high ‘overall’ scores for model importance were extracted from the PV-All, PV-BPS and PV-Human models. As the order of the important features is biased by how that importance score is calculated, when investigating such features, we opted to create a pool of high-scoring PVs from which we compared against experimental data to detect interesting candidates. The full list of important features can be found in Table S3, and the cross-referenced list of genes of high-scoring PVs that were influential for STm host-specific virulence in cattle [45] can be found in Table S16.

While features can be arranged by importance for each model, these features often vary by only a few percentage points between the host groupings, and it is only by combining information across hundreds of features that the models make a final prediction (Table S4). For example, *hdeB* is involved in acid resistance. It had an importance score of 70 % in the ‘PV, Human’ model and 30 % in the ‘PV, All’ model, indicating it could be used as a discriminant for human host selection. However, it is relatively abundant (>90 % presence) across all hosts combined and only ~2 % less frequent in the human-derived isolates. The gene *sopB* is another well studied gene that contributed to the predictions of the ‘PV, All’ and ‘PV, Human’ models (scores of 99 and 40% respectively), but, similarly to *hdeB*, it is relatively abundant across the dataset (>90 % presence across all hosts).

### Co-association of significant PVs and IGRs

Models based on IGRs consistently outperformed the PV models. In theory IGRs function as better features than PVs as they not only have PV-associated data, given they are defined by the two PVs that the IGR lies between, but they also capture any variation in that region (nucleotide and percentage length identity <90 %). To examine this, PVs that neighboured significant (i.e. used in the model) IGRs were compared against significant PVs (again those used in the models) (Tables S3, S5 and S15). The comparison showed that 584 (~58 %) of significant IGRs bordered at least one significant PV, with 272 (~27 %) bordering two significant PVs. In total, 557 (~65 %) of significant PVs bordered significant IGRs, with 108 (~12 %) associated with alternative IGR regions. The significant PVs which bordered significant IGRs included PVs presented in Table S16, such as the *hdeB* and *ybal*/*fsr*/*kefB* groups. The overlaps between significant PVs and IGRs were confirmed as statistically significant via a hypergeometric distribution test of overlap (*P*<0.01). Overall, the IGR models allow for a greater resolution of the genome (presence/absence of intergenic regions and their associated genes) than the PVs alone, which may have resulted in the noted increase in prediction accuracy.

## Discussion

### Advances in building robust *Salmonella* host and source prediction models

The ability to accurately indicate the most likely source of an isolate associated with foodborne illness based on its whole genome sequence (WGS) is valuable in outbreak investigations to narrow down potential contaminated vehicles and facilitate intervention. This is particularly important for pathogens that can originate from several animal hosts and therefore be present in a wide range of food products, such as STm, which can colonize a wide range of livestock. Routine genome sequencing of clinical isolates and of bacteria recovered from surveillance of food, animals and environments will continue to expand the datasets and improve the power of techniques applied for source attribution. Supervised ML is well suited to this task as it can process large amounts of ‘feature’ data looking for patterns that can fit to, in this case, the potential host of origin. There have been a number of models generated to look at the feasibility of different ML methods for host attribution in *Salmonella *, including application of Support Vector Machines and Random Forest. In this study, we have used a larger dataset then previously applied for STm host attribution and importantly compared multiple feature types not analysed previously, including IGRs and AMR alleles.

Particular care was taken to reduce bias in our dataset as well as potential overfitting in the resulting models. Random Forest models created from smaller feature-sets may decrease the prediction strength of individual trees (less splits), but that decrease is offset by making the model more generalizable, preventing overfitting. Therefore, in addition to implementing a clonality filter for our samples similar to the one used in the Zhang *et al.* study, we implemented a statistically based filtering step to reduce our ratio of features-to-samples to below 1. By collapsing PVs/IGRs that had the same distribution across isolates, as well as implementing a statistics-based correlation-based filtering test, we were able to reduce the features-to-samples ratio of our models to 1 : 2.

As the Centers for Disease Control estimates that, every year, *Salmonella* causes ~1.2 million infections and 26 500 hospitalizations, with STm being among the top three reported serotypes [46], analysing ~4000 isolates may not be enough to capture the full diversity of STm causing human and livestock infections. As more STm isolates are sequenced, models will need to be continually updated with new data. By automating the model building process via creating a pipeline, continual updates, refinements and further creation are possible, which will also facilitate the application of these models to source attribution/host prediction of other bacterial pathogens.

### Comparison of the performance of phylogenetic vs genomic-feature-based predictions

Phylogeny-based prediction was on a par with SNPs as features for the models, which is reassuring given that they are applying similar information. We note that the phylogeny-based predictions had quite high accuracy (~74 % Kappa statistic) and are the benchmark that ML models have to improve upon, especially for mixed clusters and ‘edge’ cases. Currently many ML models being built for source attribution favour working with whole genome MLST or equivalent SNP changes in core genes but we note, as with the results of this current study, that these are very likely to align primarily with the phylogenetic signal. Trying to separate phylogenetic signals from host assignment is arguably redundant as the two will often be tightly associated, but it is then important to determine if other features such as specific genes and intergenic regions can add further discrimination. It is logical that presence or absence of non-core genes could be discriminatory for a host as could regulation of core and accessory genes. This is supported by the fact that PVs and IGRs as features surpassed the phylogenetically and SNP-based prediction and included the capacity to correctly predict many edge cases. Moreover, it is striking that IGRs do perform better than PVs and indicate they hold more relevant information about host origin than coding sequences alone.

### Comparison of features for model building

Based on previous literature of genetic importance [10, 47–50] and approaches to host prediction models [16–18, 38, 51], SNPs, PVs and IGRs were extracted as predictors. AMR profiles were also tested as features, and while these latter models performed poorly by comparison, ongoing research is exploring relationships between AMR patterns, animal host and predicted attributed host for human isolates. Extracting features separately was preferred over a k-mer approach to reduce the number of initial predictors, i.e. to have smaller separate heterogeneous feature sets vs one very large homogenous feature set, and perform specialized filtering depending on each feature. It also allowed for easier identification of a feature deemed important to align it back into the dataset. There is also the possibility, although not attempted in this study, to combine the most important predictors from different feature sets to create new models. Additionally, though hosts such as poultry had generally more PVs and IGRs associated with them during the filtering step, there was a significant overlap of features across hosts, meaning a feature was more probably statistically associated with multiple hosts than being specific to a single host (Figs S1 and S2).

Across a wider scale of *S. enterica* serovars, Wheeler *et al.* [15] applied Random Forest to analyse allelic variation coupled with impact on gene function to identify STs of STm with a higher ‘invasiveness index’ for humans such as ST313. The training for this index is in relation to knowledge of systemic disease (in humans) across different *Salmonella* serotypes. While PVs used in our study will capture some of the same allelic variation, their method does offer a refinement of its impact at the functional level but is hard to apply. We note that a small set of ST313 were scored in our study as human, indicating the possibility of now being more likely to be transmitted between humans rather than a recent animal source.

The main finding of this study was that the most accurate models were based on IGRs and out-performed PVs even though both models had similar numbers of features, which indicates the improvement is unlikely to be due to overfitting. We interpret the higher accuracy of IGRs to be a result of not only capturing information equivalent to gene (PV) presence and absence, but also capturing nucleotide/length variation (>10 %) in IGRs. Therefore, variations of regulatory sequence-controlling PVs would, to some extent, have been captured by the resulting IGR model. Existing studies have taken note of the role of variable expression profiles and subsequent differences in virulence [10, 52], and future research will aim to define examples of such variation in this dataset, which could be investigated for their impact on the biology of STm. This level of resolution is the next frontier of phenotypic variation when considered in partnership with allelic variation [53].

We also attempted to assess if significant IGR features are more likely to cluster on the genome with significant PVs, which might indicate regulatory variation of important genes as enabling better prediction of the association with a certain host. Though a statistically significant number of important PVs bordered important IGRs and vice versa, investigating larger loci proved difficult. Using LT2 or SL1334 to map significant PVs and IGRs on a single representative genome proved a challenge, as only about a third of significant PVs and IGRs were mapped to those reference genomes. Alternatively, an analysis based on graphical genome representation is a challenge when thousands of genomes are being analysed, especially when there is considerable variation between genomes due to horizontal gene transfer and recombination-based rearrangement. Methods that utilize the variability in connectivity within the pan-genome of populations of bacterial genomes in a predictive framework are needed.

### Source prediction of human-derived STm assemblies

We envisage that the real-world use of models created on existing isolates will be regarding the prediction source and human threat for new USA STm isolates. To approximate this, an additional set of recent (2019–2022) STm farm animal-derived assemblies was obtained from Enterobase and were completely unseen from the model during training. The PV and IGR models also performed well on this dataset (Table 4), although there was a reduction in the Kappa value (Table 2). The lower performance indicates that there may be some degree of overfitting in the models. It also showcases the need to update models with new sequence data, to ensure they capture any additional genomic diversity that may not have been present during training yet or arose after the collection of the training dataset.

Obtaining an accurate breakdown of the zoonotic sources of *Salmonella* infections in the human population is difficult, especially for a generalist serovar such as STm. Our most accurate models (IGR-BPS and PV-BPS) predicted that ~45 % of the human STm isolates originated from bovine, ~40 % from poultry and~14.5 % from porcine. In comparison, the study by Zhang *et al.* [18] found that their human-derived STm isolates were attributed to zoonotic sources with the following proportions: 43 % poultry, 31 % porcine, 22 % bovine and 4 % wild birds. Their metric for an accurate livestock prediction was based on calculating Simpson’s Diversity Index values of their confidence scores, whereas ours were determined using ROC-derived thresholds. As an additional comparison, a Danish source attribution model created by Munck *et al.* [17] yielded predictions for 81 % of their human-derived *Salmonella* samples, with porcine making up the majority of predictions (69 %), followed by poultry (10 %) and cattle at <1 %. Note that their study had a much more limited set of input data (broilers *n*=34, cattle *n*=2, ducks *n*=11, layers *n*=4 and pigs *n*=159), and the heavy weighting towards porcine strains is likely to have influenced the outputs. Differences between attributed sources, beyond them being produced by a different ML model, may also indicate different levels of exposure across different continents and countries, either due to a different strain diversity or due to the presence of different transmission routes. It is planned to build an equivalent UK-Europe STm model and it will be interesting to see how the same strains are allocated in the different models to examine the impact of geography on the predictions. Moreover, it is also appreciated that human cases in particular can also be acquired from international travel or through consumption of contaminated imported food and so models will also need to take account of this.

### Caveats

The construction of all the host attribution models is limited by the metadata for each strain. Simply because a strain was isolated from a specific animal does not mean it is restricted to that species or that it has recently adapted and evolved to that host. An alternative would be to build host attribution models generated from a dataset in which each strain was tested across a variety of hosts, with more detailed data on infection (duration, severity, site of infection, etc.). However, in this case, there would be issue with animal numbers and host variation potentially biasing the data, so such infection studies may need to be on a very large and unjustifiable scale. We consider that at least the approach taken which incorporates data from thousands of isolates should reduce the ‘noise’ of such ‘false’ attribution in the training set and still allow accurate models to be generated and biologically relevant features for host assignment to be extracted and investigated. We acknowledge that with the strain information currently available it is possible that some error is introduced into our models although the extent of this error is unknown.

Construction of the human attribution model is even more limited by the metadata. Our current construction of the human threat attribution models divided our assemblies as human or not based on the host they were isolated in and were not tested in any way which would measure the infectivity in humans vs livestock. However, this model was constructed as a proof of concept and is one of the reasons a model-building pipeline was implemented, which would allow additional data to be incorporated in the future.

Though constructed with the largest dataset of STm samples so far, the models’ performance may still be limited by the distribution of hosts within the training datasets. As seen in Fig. 3, swine- and bovine-derived assemblies had more strains with reduced confidence, and the ‘second vote’, i.e. the host with the second-highest confidence score, was bovine for the swine samples and vice versa. It is notable that this confidence correlates with overall strain numbers in the training sets, so there is much more information from human and avian strains on which to build the models and apparently more confidence in the outcomes. However, that confusion may be because the bovine and swine samples, while lower in numbers, were also more phylogenetically mixed (e.g. cluster 4), especially when compared to the distinct clusters of our poultry (cluster 7) and human (cluster 8) STm samples.

The generalizability of the models is limited, as an LGOCV approach yielded variable results and performance could not be correlated with feature/model type/excluded cluster (Fig. 5). To that end, we emphasize that the models presented in this paper (as well as other models created using this pipeline) are used to test sequences that exist within the phylogeny the models were trained in. It is appreciated that the models presented in this paper are probably using a mixture of phylogenetic signals alongside biologically relevant genomic features for host prediction. Thus, it should be emphasized that the models presented in this study were generated to predict the host for current, publicly available US STm isolates, and thus are primarily intended for use on US STm isolates only. STm isolates from a different population structure will be more likely to be misattributed. As a consequence, we emphasize the importance of developing pipelines for model training and testing, as that allows the production of new ‘bespoke’ models, either to update a region’s models, or to generate models for another group of interest with appropriate strain diversity captured to aid accurate prediction.

### Important features

It is appreciated that deriving biological significance from an ML model is a challenging task. Of the many features identified as important, many may end up being classified as such because of random chance or because the model captured underlying population structure that does not necessarily translate to clustered features being directly related to an isolate’s ability to infect or colonize a specific host. At the same time, there is value in examining the most important features used in the models, as those sets may contain genes that have been associated with host adaptation. The use of Random Forest enables the top-ranked features for model selection to be defined and future work could investigate any underlying biology for host-based differences. Common to this and other studies, there is the challenge that many of the top-ranked features are ‘unknowns’, hypothetical proteins with little data available. Even when PVs are annotated, the use of Panaroo in this study’s pangenomic approach led to large and more diverse groups of PVs being clustered together, with representative sequences across the dataset.

One such example of the above was a cluster termed *ybal*/*fsr*/*kefB*. This cluster was present in all but one farm animal STm assembly but present in 58 % of human-derived STm genomes. In addition, this cluster was not localized to one specific phylogenetic subcluster, ruling out a single adaptation and expansion event in STm. Upon closer inspection, the cluster was made up of several gene subclusters of 98 % similarity. The gene subclusters were collapsed together under one PV due to Panaroo’s graph correction, which collapses neighbouring gene families if any two members between the two clusters have a 70 % similarity. The main genes making up the cluster were annotated as *fsr*, with a few annotated as *ybal*, and another as *kefB*, as well as some genes that were re-found by Panaroo. The majority of the *fsr* genes were identical to each other, whereas the *ybal*, *kefB* and ‘re-found’ genes were more diverse in terms of base pairs and length. In summary, this is clearly a complex chromosomal locus that undergoes structural variation and the exact nature of the difference between human and livestock isolates requires further investigation and is outside the scope of the models presented in this publication.

As described above, understanding these differences will require mapping the PVs onto fully assembled representative genomes of STm from different hosts for specific examples of the feature differences to be dissected. It is also appreciated that features such as PVs and IGRs can be present in different proportions in STm isolates from different hosts without a significant impact on host colonization when that feature is deleted or introduced. This can be due to: (1) the feature differential is incidental; (2) the feature impact is only really evident in the context of multiple features (polygenic) being altered; and (3) the feature plays a role in transmission to/from the host or its maintenance under real conditions, such as in the case of antibiotic resistance.

Screening of a common library of STm mutants for intestinal colonization of chickens, pigs and cattle revealed subsets of genes that share a conserved role across all hosts, but also some host-specific roles. Merging this with features predicted to influence host tropism may help to understand the potential impact of variation on strain phenotypes [54].

In conclusion, though validating the genomic features marked as important for host selection requires further analysis, our study builds upon and improves existing methods of host prediction for ML, showcases its potential advantages in accurate host-calling over phylogeny, and highlights IGRs as a genomic feature worth investigating in deconstructing the complexity of host specificity.

## Supplementary Data

Supplementary material 1
